# Treatment of pediatric epilepsy

**DOI:** 10.1002/ped4.70043

**Published:** 2026-01-29

**Authors:** Junxiao Li, Tinghong Liu, Chang Liu, Jie Deng, Shijie Wu, Suhui Kuang, Xiaotong Li, Zhirong Wei, Shuli Liang

**Affiliations:** ^1^ Functional Neurosurgery Department National Center for Children's Health Beijing Children's Hospital Capital Medical University Beijing China; ^2^ Neurosurgery Department Renji Hospital Shanghai Jiao Tong University Shanghai China; ^3^ Neurology Department National Center for Children's Health Beijing Children's Hospital Capital Medical University Beijing China; ^4^ Neurosurgery Department National Center for Children's Health Beijing Children's Hospital Capital Medical University Beijing China

**Keywords:** Anti‐seizure medications, Children, Disease‐modifying treatments, Epilepsy, Ketogenic diet, Surgery

## Abstract

Pediatric epilepsy is a neurological disorder arising from various etiologies, including structural, genetic, immune, infectious, metabolic, and unknown causes. Anti‐seizure medications remain the primary treatment; however, in cases of drug‐resistant epilepsy, surgical interventions, ketogenic diet, and emerging therapies have become increasingly effective options. Disease‐modifying treatments, such as antisense oligonucleotides and adeno‐associated virus–mediated gene replacement, have shown promise in some epilepsy treatments, with early trials reporting moderate seizure reduction. Minimally invasive surgical approaches, including magnetic resonance–guided laser interstitial thermal therapy, have also demonstrated favorable outcomes, showing a 68% seizure‐free rate at 2 years in the largest pediatric series. Although the ketogenic diet is effective in some patients, demonstrating superiority over conventional management for >50% seizure reduction, long‐term use may be associated with metabolic risks; careful monitoring is warranted. Future treatment strategies are expected to emphasize personalized medicine through the integration of genetic, electrophysiological, and neuroimaging data to optimize therapeutic decision‐making and enable targeted interventions based on the underlying etiology.

## Graphical abstract

Anti‐seizure medications are the first‐line treatment for the vast majority of children with epilepsy, with the advantages of non‐invasive wide adaptability. Surgery is the main treatment for drug‐resistant epilepsy and lesion‐related epilepsy, which can cure some cases of epilepsy in children. A ketogenic diet is often an add‐on therapy. Gene therapy and disease modification therapy may bring new hope to pediatric epilepsy.

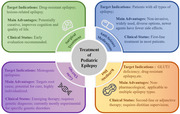

## INTRODUCTION

Epilepsy, a chronic neurological disorder characterized by abnormal neuronal hyperexcitability in the brain, represents a critical public health challenge in pediatrics due to its recurrent seizures and profound effects on neurodevelopment.^1^ Globally, epilepsy affects approximately 50 million individuals—children constitute a high‐risk population and experience an estimated 10.5 million cases worldwide.^2^ Epidemiological studies report an annual incidence ranging from 41 to 187 per 100 000 children,^3^ with age‐dependent peaks observed during infancy (<3 years) and early adolescence (10–14 years). Notably, according to the World Health Organization Global Burden of Disease Report 2023,^4^ prevalence in low‐income countries (10.3 per 1000) substantially exceeds that in high‐resource settings (5.1 per 1000). This disparity is largely attributed to inadequate perinatal care, limited management of central nervous system infections, and socioeconomic inequities.^4^

The burden of epilepsy extends beyond its clinical manifestations. Pediatric epilepsy is associated with an increased risk of premature mortality and disruption of neurocognitive development.^2^ Recurrent seizures and prolonged exposure to anti‐seizure medications (ASMs) impair synaptic plasticity, resulting in learning disabilities, memory deficits, and executive dysfunction.^5^ Children with persistent seizures demonstrate the greatest susceptibility to cognitive impairment, which is correlated with delayed intellectual and motor development. These neurodevelopmental deficits frequently coexist with psychosocial challenges, including social isolation, low self‐esteem, and increased risks of anxiety and depression. Early intervention, whether pharmacological or surgical, has demonstrated efficacy in mitigating these risks and thus underscores the importance of timely therapeutic strategies.

Recent advances in neuroscience and molecular biology have transformed the management of pediatric epilepsy. Pharmacological progress includes the development of third‐generation ASMs, which provide improved efficacy with reduced neurocognitive adverse effects. Concurrent advances in genetic diagnostics and therapeutics have enabled personalized treatment paradigms tailored to specific etiologies.

Nonpharmacological therapies have also undergone considerable evolution. Neuromodulation technologies, ablative surgical approaches, and gene‐editing therapies are redefining the therapeutic landscape for patients with drug‐resistant epilepsy (DRE).^6^, ^7^, ^8^, ^9^ For instance, closed‐loop neurostimulation systems and magnetic resonance–guided laser interstitial thermal therapy (MRgLITT) have demonstrated substantial improvements in seizure control among patients with DRE.^10^, ^11^ This review synthesizes recent evidence‐based advances in pediatric epilepsy therapeutics to inform optimized clinical practice and guide future research directions.

### ETIOLOGY AND CLASSIFICATION OF EPILEPSY

Epilepsy comprises a group of disorders with diverse etiologies. A structural etiology is defined by abnormalities visible on magnetic resonance imaging (MRI) or computed tomography that are considered the likely cause of a patient's seizures. Structural etiologies include tumors, vascular disorders, hippocampal sclerosis, and acquired conditions such as stroke, trauma, and infection, as well as genetically determined abnormalities. Genetic etiologies encompass single‐gene or multigene variants, copy number variations, chromosomal abnormalities, and mitochondrial gene defects. Genetic epilepsy directly originates from a known or presumed genetic mutation, in which seizures constitute a core feature of the disorder. Immune, infectious, and metabolic epilepsies directly arise from immune‐mediated, infectious, or metabolic disorders, respectively, in which seizures are a central manifestation. These conditions may also result in acute provoked seizures or chronic unprovoked epilepsy. When the cause of epilepsy has not been identified, it is classified as “unknown”.^12^

Epileptic seizures encompass a wide range of clinical types. According to the International League Against Epilepsy (ILAE) 2025 Classification of Epileptic Seizures, seizures are categorized as focal, generalized, unknown, or unclassified.^13^ Focal seizures are those that originate within networks limited to one cerebral hemisphere. Generalized seizures originate at some point within, and rapidly engage, bilaterally distributed networks; they may include cortical and subcortical structures but do not involve the entire cortex. When information is available to characterize certain seizure features but remains insufficient to permit classification as focal or generalized, seizures are categorized as “unknown”. When no information is available to characterize the seizure, but the clinician is confident that the event represents an epileptic seizure, the “unclassified” designation is used.^13^

### ANTI‐SEIZURE MEDICATIONS

ASMs represent the initial and most important treatment option for most patients with epilepsy. Monotherapy is preferred as the first‐line approach; rational polytherapy may be considered when necessary. ASMs may also be administered concurrently with surgical interventions and ketogenic diet therapy. Selection of ASMs should consider multiple factors, including seizure type and epilepsy syndrome, patient age and sex, comorbid physical conditions, mechanisms of action, pharmacokinetic properties, potential drug–drug interactions, and medication cost and accessibility.

Since phenobarbital was introduced for epilepsy treatment in 1912, more than 30 ASMs have been approved and marketed. Clinically, first‐generation and second‐generation ASMs have been widely used and have benefited many patients with epilepsy (Table 1). First‐generation ASMs include phenobarbital, phenytoin, valproate, ethosuximide, clonazepam, and carbamazepine. Representative second‐generation ASMs include topiramate, oxcarbazepine, levetiracetam, lamotrigine, gabapentin, and zonisamide. Over the past decade, numerous novel ASMs have been developed.^14^ Lacosamide and perampanel are indicated for focal seizures and generalized tonic–clonic seizures.^14^, ^15^ Eslicarbazepine, brivaracetam, and cenobamate are approved for partial‐onset seizure treatment. Evidence supporting its efficacy in children aged 6–18 years with drug‐resistant focal epilepsy remains insufficient.^16^, ^17^, ^18^, ^19^, ^20^, ^21^, ^22^, ^23^ Cannabidiol demonstrates significant antiseizure efficacy and has been associated with improved sleep quality.^24^, ^25^, ^26^ However, the pediatric evidence base remains limited and largely relies on open‐label studies, retrospective analyses, or extrapolation from adult trials in those ASMs.^14^, ^15^, ^18^, ^20^, ^21^, ^23^, ^26^

**TABLE 1:** Mechanisms of action and indications of anti‐seizure medications

ASMs	Mechanisms of action	Indications	
		With RCT evidence	Without RCT evidence
First‐generation			
Phenobarbital	Enhancing GABA	Focal seizure	GTCS, myoclonic seizure
Phenytoin	Blocking sodium channels	Focal seizure	GTCS
Valproate	Blocking sodium channels Enhancing GABA Blocking T‐type calcium channels	Focal seizure, absence seizure −	GTCS, myoclonic seizure, other seizures
Ethosuximide	Blocking T‐type calcium channels	Absence seizures	−
Clonazepam	GABA‐mediated chloride channel	Generalized seizures	−
Carbamazepine	Blocking sodium channels	Focal seizure	GTCS
Second‐generation			
Topiramate	Blocking sodium channels AMPA/glutamate antagonism Enhancing GABA	Focal seizure, GTCS, LGS	−
Oxcarbazepine	Blocking sodium channels	Focal seizure, GTCS	Absence seizure
Levetiracetam	Binding synaptic vesicle protein 2A	Focal seizure, myoclonic seizure, GTCS	Absence seizure
Lamotrigine	Blocking sodium channels	Focal seizure, GTCS, LGS	Absence seizure, myoclonic seizure
Gabapentin	Binding α2δ calcium channel subunit	Focal seizure	−
Zonisamide	Blocking sodium channels Blocking T‐type calcium channels	Focal seizure	GTCS, absence seizure, myoclonic seizure
Third‐generation			
Lacosamide	Blocking sodium channels	Focal seizure	−
Perampanel	AMPA/glutamate antagonism	Focal seizure with or without secondarily generalized seizures	GTCS in patients with idiopathic generalized epilepsy
Vigabatrin	Enhancing GABA	−	Infantile epileptic spasm syndrome, refractory complex focal seizures
Eslicarbazepine	Blocking sodium channels	Focal seizure	Drug‐resistant focal epilepsy
Brivaracetam	Binding synaptic vesicle protein 2A	Focal seizure	Focal seizure
Ganaxolone	Enhancing GABA	−	Seizures associated with CDKL5 deficiency disorder
Felbamate	N‐methyl‐D‐aspartate antagonism Enhancing GABA Blocking sodium channels Blocking calcium channels	−	Adjunctive therapy in focal and generalized seizures associated with LGS
Cenobamate	Blocking sodium channels Enhancing GABA	Partial‐onset seizures in an adult patient	−
Clobazam	Enhancing GABA	Adjunctive treatment in LGS	Adjunctive treatment in Dravet syndrome
Cannabidiol	G protein‐coupled receptor 55 and transient receptor potential vanilloid 1 channels modulator Equilibrative nucleoside transporter 1 inhibitor	LGS, Dravet syndrome, tuberous sclerosis	−

Abbreviations: AMPA, α‐amino‐3‐hydroxy‐5‐methyl‐4‐isoxazolepropionic acid; ASMs, anti‐seizure medications; GABA, γ‐aminobutyric acid; GTCS, generalized tonic‐clonic seizure; LGS, Lennox‐Gastaut Syndrome; RCT, randomized controlled trial.

In addition, several ASMs are also indicated for the treatment of specific types of epilepsy. Clobazam is an established adjunctive therapy for Lennox–Gastaut syndrome (LGS) in children aged >2 years, with efficacy supported by high‐quality randomized controlled trials (RCTs).^27^, ^28^ Vigabatrin is commonly prescribed for infantile epileptic spasm syndrome and as adjunctive therapy for refractory complex partial seizures, particularly in conditions such as tuberous sclerosis complex (TSC).^29^, ^30^, ^31^ Ganaxolone has been confirmed to have efficacy and tolerability in seizures associated with cyclin‐dependent kinase‐like 5 deficiency disorder.^32^, ^33^, ^34^ Felbamate is primarily used as adjunctive therapy for LGS.^35^ However, high‐quality long‐term data of efficacy and safety in children remain limited in those three ASMs.^31^, ^34^, ^35^

### SURGICAL TREATMENTS

Recent nationwide data from the United States indicate a substantial increase in the volume of pediatric epilepsy surgeries.^36^ The Surgical Therapies Commission of the ILAE recommends prompt surgical evaluation after confirmation of DRE.^37^ Surgical referral should be considered for patients who remain seizure‐free on one or two ASMs but harbor lesions in the non‐eloquent cortex.^37^ Among children aged <3 years, emerging clinical evidence indicates favorable outcomes after surgical treatment; delayed referral has been associated with worse prognoses.^38^, ^39^ There is increasing clinical evidence to support surgical intervention for prognostic improvement.

Neuropsychological function in children with DRE often exhibits a declining trajectory.^39^, ^40^ Patients with postoperative seizure freedom not only experience arrest of this decline but also demonstrate progressive neuropsychological recovery. Notably, individuals who achieve ASM withdrawal after surgery show superior neuropsychological outcomes compared with patients who continue medication treatment.^40^, ^41^ To promptly arrest and reverse neuropsychological deterioration, early surgical intervention is strongly recommended, followed by timely ASM discontinuation in seizure‐free cases. Current surgical approaches for epilepsy include resective surgery, neuromodulation, ablative surgery, and disconnective surgery.^9^ The following sections describe recent advances in these surgical modalities based on updated literature.

### Resective surgery

Removal of the epileptogenic brain region has represented the most definitive approach for seizure control in patients with DRE for over a century.^8^, ^9^ It remains the most effective curative option; advances in neurosurgical techniques and epilepsy localization technologies have substantially improved its safety and efficacy.^8^, ^42^

When the epileptogenic focus is clearly localized to a brain region that can be safely resected, resective surgery offers the highest likelihood of seizure freedom in children with DRE and should be considered the preferred treatment. The superiority of epilepsy surgery over medical management in pediatric DRE was demonstrated in an RCT.^43^ At the 12‐month follow‐up, seizure freedom was achieved in 44 surgical patients (77%) compared with four patients receiving medical management alone (7%) (*P* < 0.001). This single‐center study showed that children and adolescents who underwent resective surgery had significantly higher seizure‐free rates and better behavioral and quality‐of‐life outcomes than those exclusively treated with medical therapy.^43^ Recent clinical evidence further indicates that resective surgery is associated with favorable outcomes.^38^, ^39^ From a prognostic perspective, early attainment of seizure freedom permits brain development to proceed without the adverse effects of seizures. A very recent study indicated that epilepsy surgery can lead to meaningful adaptive improvements in children with DRE, including those with severe neurological impairment, particularly among patients who achieve seizure freedom.^44^

Recently, to address challenges associated with the identification and resection of MRI‐negative or deep‐seated epileptic foci, a neuro‐robotic navigation system specifically designed for these procedures was introduced. In the neuro‐robotic group, a robotic laser device precisely delineated lesion boundaries to guide surgical resection and localized the deepest points to define the margins of the epileptogenic focus. The results demonstrated efficacy comparable to conventional neuronavigation in MRI‐positive patients (Engel class I rate: 71.4% vs. 100%, *P* = 0.255), whereas superior outcomes were observed in MRI‐negative focal cortical dysplasia cases (Engel class I rate: 88.2% vs. 50%, *P* = 0.0439). These findings indicate improved surgical outcomes, particularly in the resection of MRI‐negative or deep‐seated epileptic foci.^45^ The combination of favorable seizure outcomes, increased life expectancy, and improved quality of life supports resective surgery as the treatment of choice for children with DRE.

### Neuromodulation

Although seizure freedom is rarely achieved with neuromodulation, reductions in seizure frequency and severity are commonly reported.^7^, ^42^ Beyond symptomatic control, emerging evidence suggests that chronic neuromodulation can promote progressive neurophysiological reorganization within epileptic networks.^46^, ^47^ Mechanisms such as retuning of thalamocortical circuits, plasticity‐mediated shifts in neuronal excitability thresholds, and long‐term adaptive changes after sustained stimulation are under exploration. These observations position neuromodulation as a palliative intervention and potential intermediate disease‐modifying strategy capable of gradually altering network dynamics. Three neuromodulation modalities are currently used in clinical practice: vagus nerve stimulation (VNS), deep brain stimulation (DBS), and responsive neurostimulation (RNS). A large meta‐analysis showed that adjunctive VNS leads to >50% seizure reduction in more than half of pediatric patients. A later age at epilepsy onset and fewer ASMs administered before VNS implantation have been strongly associated with more favorable steady‐state seizure outcomes.^46^ Consequently, children with epilepsy who are not candidates for resective surgery may benefit from early consideration of VNS, rather than postponement until multiple prolonged drug trials or definitive confirmation of drug resistance.

A systematic review comparing DBS and RNS targeting thalamic nuclei reported a higher treatment response rate with DBS than with RNS (76.8% vs. 57.9%).^47^ Although this difference did not reach statistical significance, the findings suggest an advantage for DBS targeting the anterior thalamic nucleus in the management of focal epilepsy. These results indicate clinically meaningful differences in efficacy that may inform clinical decision‐making toward anterior thalamic nucleus DBS for pediatric focal epilepsy; higher‐quality evidence is required to confirm superiority over RNS with respect to long‐term seizure control and neuropsychological outcomes. The field is increasingly moving toward biomarker‐guided neuromodulation. The identification of electrophysiological or neuroimaging biomarkers predictive of treatment response is particularly relevant for pediatric patients with diffuse or multifocal epileptogenic networks. Goals include optimizing target selection and stimulation parameters.

In children with DRE, RNS has demonstrated meaningful therapeutic efficacy. A retrospective case series evaluated the feasibility of closed‐loop RNS targeting the bilateral centromedian thalamus in 16 children with DRE.^48^ At a median follow‐up of 1.3 years, 62% of patients achieved ≥50% reduction in seizure frequency. Notably, two patients maintained seizure freedom, suggesting the possibility of sustained neuromodulatory effects. The centromedian thalamus is a promising therapeutic target for RNS in pediatric DRE, and this intervention shows feasibility.

In LGS, the centromedian thalamus has been proposed as a therapeutic target. The landmark ESTEL trial, a prospective double‐blind randomized controlled study, systematically evaluated the efficacy of bilateral centromedian thalamic DBS in 20 young adults with LGS.^49^ A median reduction of 46.7% in overall seizure frequency was observed at study completion. These results demonstrate the feasibility and therapeutic potential of centromedian thalamic DBS in young adult patients with LGS, while also providing an important foundation for future investigations of DBS‐based interventions in pediatric populations with LGS.

Regarding combination neuromodulation therapies, a partially randomized patient‐preference trial was conducted in children who achieved below 50% reduction in seizure frequency ≥1 year after VNS implantation.^50^ Seven underwent add‐on DBS implantation; seizure outcomes were compared with those of 11 children exhibiting similar baseline demographics and seizure characteristics who continued VNS therapy alone. Among patients receiving combination therapy, a significant reduction in seizure frequency was observed (12.3% in the VNS‐only group vs. 51.9% in the DBS–VNS group), along with a reduction in mean frequency of the most debilitating seizure type at 1 year. Comparison of Seizure Severity Questionnaire scores demonstrated a significant reduction in the most bothersome seizure type; no significant change was observed in the most severe seizure type.^50^

A case series evaluated combined VNS and RNS targeting the bilateral centromedian thalamus in seven pediatric patients. Twenty‐four‐hour intracranial electroencephalography analyses demonstrated reductions of 75%–99% in disabling seizures across all patients at a median follow‐up of 1.2 years. The study confirmed the safety of dual‐device therapy; no device–device interactions or serious adverse events were reported.^51^ These findings highlight the potential of multimodal neuromodulation strategies for pediatric refractory epilepsy.

### Ablative surgery

Ablative surgical approaches encompass two principal modalities: thermocoagulation and ionizing ablation. Thermocoagulation, which has served as a cornerstone minimally invasive technique in epilepsy surgery for more than six decades, induces protein denaturation and tissue vaporization through localized hyperthermia and includes focused ultrasound, radiofrequency thermocoagulation (RF‐TC), and MRgLITT. Ionizing ablation is performed using gamma knife technology. Given the currently limited clinical application of focused ultrasound and ionizing ablation techniques, including gamma knife, the following discussion focuses on stereotactic radiofrequency ablation and laser‐based therapies.^11^, ^52^

RF‐TC is a minimally invasive neurosurgical technique that utilizes radiofrequency energy to create targeted lesions in regions associated with seizure generation.^11^, ^53^ By selectively disrupting epileptogenic neural pathways, RF‐TC modulates pathological circuits while preserving adjacent functional tissue. Since its introduction in the 1980s, this technique has served as a valuable alternative to resective surgery in patients with DRE, particularly when lesions are located within eloquent brain regions. The integration of stereoelectroencephalography has substantially enhanced RF‐TC by enabling real‐time electrophysiological guidance, thus improving target localization and therapeutic precision. However, evidence supporting pediatric applications remains limited.^54^

A multicenter cohort study (*n* = 46) evaluating RF‐TC for epilepsy showed responder rates (≥50% seizure reduction) of 69.6% at 1 month and 73.5% at 1 year after the procedure.^55^ The study suggested that RF‐TC is safe and effective in children, but applicability was limited by stringent eligibility criteria—21.3% of screened patients met protocol requirements. More refined patient‐selection frameworks are needed.

In complex neurogenetic disorders, RF‐TC has demonstrated considerable therapeutic potential. A recent study (*n* = 9) detected complete remission in 88.9% (8/9) of patients at 6 months; 85.7% (6/7) maintained seizure freedom for more than 1 year.^56^ These outcomes exceed conventional pharmacological efficacy rates, positioning RF‐TC as a promising option for refractory cases.

Despite encouraging results, current evidence is constrained by small sample sizes, heterogeneous protocols, and limited longitudinal follow‐up. For instance, Bottan et al.^54^ reported inconsistent definitions of treatment response across studies, which complicates cross‐study comparisons. Large‐scale RCTs with standardized outcome measures are required to define the role of RF‐TC in pediatric epilepsy management. Cost–benefit analyses comparing RF‐TC with neuromodulation therapies should be prioritized to inform clinical decision‐making.

To mitigate the risks associated with resective surgery, including infection, hemorrhage, and prolonged recovery, MRgLITT has emerged as a minimally invasive alternative for DRE treatment, particularly in cases involving deep‐seated or eloquent brain lesions. This technique uses real‐time magnetic resonance thermometry to deliver precise thermal energy through a stereotactically implanted laser fiber, inducing coagulative necrosis within epileptogenic zones while minimizing collateral injury. The largest pediatric MRgLITT series reported to date (*n* = 225) demonstrated that among patients with ≥24 months of follow‐up, 47.2% achieved an Engel Class I outcome (seizure‐free), and 86.1% achieved Engel Class I or II (favorable outcome). Permanent neurological deficits were observed in only 1.33% of patients, supporting a favorable safety profile.^57^ However, concerns regarding long‐term durability persist. Among 49 patients with at least 12 months of follow‐up, Engel class I outcomes declined from 51.0% at 1 year to 42.1% at 4 years.^57^

In comparison with open epilepsy surgery, a propensity score–matched noninferiority study directly evaluated MRgLITT (*n* = 185) and open surgical resection (*n* = 185). At 1 year, seizure freedom rates were significantly higher in the open surgery group (61.6% vs. 48.1%), particularly among patients with malformations of cortical development (76.7% vs. 43.3%, *P* < 0.001). Nevertheless, MRgLITT demonstrated substantial safety advantages, with lower procedural morbidity (10.8% vs. 29.2%) and a shorter median hospital stay (3.1 vs. 7.2 days).^58^ Although MRgLITT offers meaningful safety benefits, its long‐term efficacy—particularly regarding sustained seizure freedom—may be less robust than that of conventional open surgery.

Although MRgLITT provides significant safety advantages and minimal invasiveness, it may not fully replace open resective surgery for lesions requiring extensive cytoreduction.^59^ This approach is particularly advantageous for deep‐seated or bilateral epileptogenic zones, where open resection carries a higher risk of functional impairment.^59^, ^60^ The capacity to target multiple regions in a repeatable manner enables staged ablation strategies for multifocal epilepsy; this option is not feasible with conventional surgery.^59^, ^60^, ^61^ Despite favorable safety and efficacy profiles, a substantial gap persists in the literature regarding direct, long‐term head‐to‐head comparisons between MRgLITT and conventional craniotomy. The absence of comprehensive comparative data highlights the need for further studies to better define the role of MRgLITT in pediatric epilepsy surgery, particularly concerning its long‐term effectiveness relative to more invasive surgical approaches.^61^, ^62^, ^63^

### Disconnective surgery

Disconnective surgery, a key component of functional neurosurgery, achieves seizure control via targeted interruption of epileptic network pathways while preserving overall anatomical structure. Disconnective procedures are broadly categorized into radical disconnections (hemispherectomy and lobar disconnection) and palliative disconnections (e.g., corpus callosotomy [CCT]).^64^

Hemispherotomy is indicated for the treatment of patients with medically intractable epilepsy localized in one cerebral hemisphere.^65^ By completely interrupting intrahemispheric and interhemispheric propagation pathways, hemispherotomy has become a cornerstone surgical option for pediatric DRE, representing approximately 20%–40% of pediatric epilepsy surgeries.^66^

Hemispherotomy can effectively control seizures in carefully selected pediatric patients by functionally disconnecting the entire affected cerebral hemisphere. A retrospective study analyzing postoperative outcomes in 457 children who underwent hemispherotomy identified seizure freedom in 75% of patients. No statistically significant differences in seizure reduction or postoperative recurrence rates were observed between vertical and lateral hemispherotomy techniques in this cohort.^66^ A separate meta‐analysis of 686 cases demonstrated that vertical hemispherotomy was associated with superior seizure control and lower recurrence rates relative to lateral hemispherotomy, with statistically significant differences; these findings were supported by an additional retrospective cohort study.^67^, ^68^ Such conflicting results warrant high‐quality, comparative studies to clarify surgical efficacies and inform clinical decision‐making.

Early cohort studies identified etiology as a critical determinant of postoperative functional outcomes in children.^69^ Developmental etiologies such as hemimegaloencephaly have been associated with less favorable motor and language outcomes. More recent studies suggest that prolonged epilepsy duration also negatively affects postoperative language function.^70^ Later seizure onset and timely discontinuation of ASMs after hemispherotomy are associated with improved intellectual developmental outcomes.^71^ These findings refine preoperative evaluation criteria and emphasize the importance of postoperative ASM management. Early ASM withdrawal remains the only modifiable factor consistently associated with improved cognitive development in this patient population.

Although its clinical application has declined, CCT disrupts bilateral synchronization of epileptic activity by severing interhemispheric fibers of the corpus callosum, thus reducing the frequency and severity of generalized seizures. This procedure is primarily indicated for patients with drug‐resistant generalized epilepsy, especially those exhibiting LGS or epileptic encephalopathy accompanied by drop seizures. Open microsurgical CCT has long represented the standard surgical approach for drug‐resistant generalized epilepsy; however, its substantial invasiveness and prolonged recovery have prompted investigation of less invasive alternatives.^72^, ^73^ In recent years, MRgLITT has emerged as a promising technique. Multiple comparative studies of MRgLITT and open CCT have demonstrated comparable seizure outcomes, without statistically significant differences.^74^, ^75^

A multicenter prospective observational comparative study in the United States evaluated open, MRgLITT, and endoscopic approaches to CCT. The results demonstrated that CCT remains an effective intervention regardless of surgical technique; complication rates were within acceptable ranges across all approaches. MRgLITT was associated with substantially reduced intraoperative blood loss, shorter hospital stays, and lower complication rates.^76^ However, this technique is hindered by longer operative times and higher costs, which impose substantial financial burdens on families and increased demands on clinical resources.^54^ Combined therapy using CCT and neuromodulation has gradually been introduced into clinical practice. CCT reduces bilateral synchronization through anatomical disconnection, whereas neuromodulation suppresses epileptic network activity at a functional level by modulating excitability within the brainstem, limbic system, and cerebral cortex. The complementary mechanisms of these approaches provide a theoretical rationale for combined application. A multicenter study indicated that among patients with LGS who underwent CCT after VNS, at least 50% reduction in drop attacks and other seizure types was achieved in 83% and 60% of patients, respectively.^77^ These findings suggest new directions for improving seizure control and quality of life. Nevertheless, the optimal timing of combination therapy and potential adverse effects associated with concurrent use require further evaluation through long‐term follow‐up studies.

### KETOGENIC DIET

The ketogenic diet—characterized by high fat, low carbohydrate, and moderate protein intake—mimics a fasting metabolic state and induces hepatic ketogenesis, with production of β‐hydroxybutyrate, acetoacetate, and acetone.^78^ These ketones replace glucose as the primary energy source, modulating neuronal excitability and suppressing abnormal epileptic discharges.^79^

Although the ketogenic diet has shown limited efficacy in adults, increasing clinical evidence supports its role as an adjunctive therapy for seizure reduction in children with DRE. A meta‐analysis conducted in the United States comparing three dietary therapies with usual care demonstrated that the ketogenic diet was superior to conventional management in achieving a >50% reduction in seizure frequency.^78^ Multiple systematic reviews, despite reliance on low‐quality evidence, have reported improved seizure control with ketogenic diet therapy in early‐onset epilepsy and DRE.^79^, ^80^, ^81^ The ketogenic diet may hold clinical value compared with usual care for seizure management. A European multicenter, open‐label, extended randomized clinical trial comparing ketogenic diet therapy with additional ASMs among infants with DRE identified no significant differences in efficacy or tolerability between the two approaches; the ketogenic diet demonstrated a favorable safety profile.^82^ Thus, the ketogenic diet should be considered a viable alternative for children who do not respond to ASMs.

Long‐term ketogenic diet therapy requires careful metabolic monitoring. A multicenter study linked prolonged ketogenic diet use to hypercalcemia; multiple reports have documented adverse effects, including vomiting, constipation, and diarrhea.^78^, ^83^ In summary, short‐term ketogenic diet therapy effectively reduces seizure frequency and provides symptomatic benefit, but vigilant monitoring is necessary to minimize the risk of long‐term complications.

### DISEASE‐MODIFYING THERAPIES

Advances in etiological detection technologies have increased the identification of developmental and epileptic encephalopathies in children previously considered to have unexplained epilepsy. For many of these patients, surgical intervention remains impractical due to diffuse brain involvement that is not confined to discrete anatomical regions. Current ASMs primarily offer symptomatic control and do not fundamentally alter disease progression. Although neuromodulation and ketogenic diet therapy are often used as adjunctive treatments, they rarely yield seizure freedom. Consequently, rapidly evolving gene‐based disease‐modifying therapies have emerged as promising approaches to address underlying disease mechanisms.

Disease‐modifying therapy refers to treatment strategies that target the pathophysiological mechanisms underlying disease progression, thereby altering the clinical course of disease. In contrast to purely symptomatic interventions, a defining feature of disease‐modifying therapy is the capacity to interfere with or potentially reverse disease progression, with sustained benefit even after treatment discontinuation. Emerging approaches can be broadly categorized as follows: (1) ion channel correction through antisense oligonucleotide (ASO)‐mediated transcript rescue or adeno‐associated virus (AAV)‐based gene supplementation, as exemplified in Dravet syndrome^84^; (2) precision metabolic modulation (e.g., cerebral folate or biotinidase deficiency treatment)^85^; and (3) pathway‐level interventions targeting broader mechanisms, including GABAergic interneuron development, mitochondrial bioenergetics, or glutamatergic receptor regulation. This framework helps to distinguish between true disease‐modifying therapies and interventions that primarily reduce seizure burden without altering long‐term cognitive or structural outcomes.

### Targeted gene therapy for the ion channel

Gene therapy directly targets genetic defects through strategies such as ASO–mediated splicing modulation and AAV–mediated gene replacement; it has emerged as a transformative approach for monogenic epilepsy treatment. By restoring ion channel function, rebalancing neural network excitability, or suppressing aberrant signaling pathways, gene therapy interventions offer precise strategies that address the underlying pathophysiology of developmental epileptic encephalopathies.

For instance, STK‐001 (ASO‐22), an ASO targeting the sodium channel, voltage‐gated, type I α subunit (*SCN1A*)‐mutated Dravet syndrome, has entered clinical trials. In the United States, these trials primarily enroll patients with Dravet syndrome aged 2–18 years to evaluate the efficacy and safety of single‐ and multiple‐dose escalation regimens of STK‐001. To date, 62 participants have received treatment, and preliminary findings indicate a moderate reduction in seizure frequency.^86^ Additional studies and comprehensive data analyses are required to validate these results. Preclinical investigations have also examined the therapeutic effects of a novel ASO (ASO‐84) in *SCN1A*‐mutated Dravet syndrome models.^87^ These studies demonstrated restoration of action potential firing to 92% of wild‐type levels, sodium current density from 50% to 95%, and a 1.8‐fold increase in GABAergic synaptic transmission frequency in cortical parvalbumin‐positive interneurons. Electrophysiological findings indicate an antiepileptic mechanism mediated by rebalancing inhibitory network activity, which supports ASO‐mediated upregulation of Na_v_1.1 as a promising therapeutic strategy for Dravet syndrome. Despite encouraging preclinical results, translation to human application requires further investigation.^87^

An additional study evaluated an AAV‐mediated gene replacement strategy to restore β1 subunit expression in ASO targeting the sodium channel, voltage‐gated, type I β subunit (*SCN1B*)‐mutated Dravet syndrome models.^88^ Therapy was administered at two developmental stages: neonatal (postnatal day 2, P2) and juvenile (postnatal day 10, P10). Neonatal administration considerably extended lifespan and reduced seizure burden, whereas juvenile administration—even at doubled doses—conferred no therapeutic benefit. Early intervention timing is critical in gene therapy; developmental windows should be prioritized in clinical trial design. The long‐term consequences and potential risks associated with neonatal administration require careful evaluation.^88^

In a study focused on focal cortical dysplasia type II, a potassium channel transgene was engineered under the control of the CAMK2A promoter, which preferentially drives expression in principal neurons. The construct was packaged into an AAV serotype 9 (AAV9) vector and injected into dysplastic cortical regions; this approach achieved a 64% reduction in seizure frequency.^89^ This work represents an early example of subtype‐specific neuromodulation and provides a conceptual framework for DRE treatment. However, only 22% of dysplastic neurons were transduced in the study by Almacellas Barbanoj et al.,^89^ underscoring the need for improved AAV tropism or enhanced promoter specificity.

### Pyridoxine supplementation therapy

The treatment of pyridoxine‐dependent epilepsy (PDE) with pyridoxine represents a paradigmatic model of disease‐modifying therapy for metabolically targeted etiologies. PDE is a rare genetic disorder caused by pathogenic variants in the *ALDH7A1* gene. Early clinical manifestations may include hypothermia, irritability, and sleep disturbance; some patients initially present without seizures, which frequently leads to misdiagnosis.^90^

The *ALDH7A1* gene is involved in lysine degradation, and patients with PDE exhibit accumulation of biomarkers (e.g., 5‐α‐aminoadipic semialdehyde, piperideine‐6‐carboxylate, and pipecolic acid). Pyridoxine supplementation substantially reduces concentrations of these metabolites and improves clinical outcomes. An observational study of 55 children, stratified into four treatment groups, demonstrated that patients who received pyridoxine as the initial therapeutic strategy achieved superior seizure control and more favorable long‐term neurodevelopmental outcomes compared with those who received delayed pyridoxine or ASMs.^91^ Early pyridoxine therapy initiation is critical to reduce seizure risk and mitigate neurodevelopmental impacts.

A recent study in China identified five cases of PDE presenting with infantile epileptic spasm syndrome. All five patients harbored eight distinct pathogenic variants in the *ALDH7A1* gene. Although seizure control improved with vitamin B6 therapy, varying degrees of developmental delay persisted.^92^ To clarify mechanisms underlying neurodevelopmental impairment, Yan et al.^93^ conducted an animal study demonstrating that the pathology was attributable to impaired *de novo* pyrimidine biosynthesis, rather than pyridoxine deficiency itself. Pyrimidine supplementation rescued abnormal neurodevelopment and cognitive deficits in *ALDH7A1*‐deficient mice.

### Mammalian target of rapamycin inhibitors

TSC arises from hyperactivation of the mammalian target of rapamycin (mTOR) pathway secondary to deficiencies in hamartin and tuberin. mTOR inhibitors, including everolimus and sirolimus, are now used in clinical practice. The EXIST‐3 trial provided high‐quality evidence supporting the efficacy and safety of everolimus in TSC, demonstrating robust seizure reduction.^94^ In contrast, an RCT indicated that sirolimus was ineffective for seizure control and was associated with adverse events.^95^

Although the EXIST‐3 trial excluded children aged <2 years, recent retrospective studies have supported the efficacy and safety of everolimus and sirolimus among such children.^96^, ^97^, ^98^ Beyond therapeutic applications, mTOR inhibitors are under investigation as preventive strategies. A phase I trial confirmed the safety and tolerability of presymptomatic sirolimus prophylaxis among infants with TSC and demonstrated superior outcomes compared with untreated cohorts.^99^ A phase II prospective trial is ongoing.

In summary, disease‐modifying therapies hold substantial promise for refractory pediatric epilepsy treatment, particularly involving well‐defined genetic or metabolic etiologies. A concise overview of the current evidence base for these promising but investigational approaches is shown in Table 2. The quality of evidence supporting these approaches generally remains limited, underscoring the need for larger, rigorously designed clinical trials and long‐term follow‐up to establish their roles in routine clinical practice.

**TABLE 2:** Summary of key evidence for disease‐modifying therapy in pediatric epilepsy

Therapy	Target/ Mechanism	Key evidence	Sample size	Main efficacy	Safety notes
STK‐001 (ASO)	SCN1A (Dravet syndrome)	Phase I/IIa trial (Ongoing)	62 participants (2–18 years)	Moderate seizure reduction (preliminary)	Ongoing evaluation; preliminary safety
AAV‐SCN1B	SCN1B (Dravet syndrome)	Preclinical (mouse model)	Animal study	Lifespan extension, seizure burden reduction (neonatal admin only)	Neonatal timing critical; long‐term safety needs evaluation
AAV‐potassium channel	Focal cortical dysplasia	Preclinical (mouse model)	Animal study	64% seizure frequency reduction	Low transduction efficiency in human cells noted
Everolimus	mTOR (TSC)	Phase III RCT (EXIST‐3)	∼150 (pediatric subset)	Significantly greater responder ratevs placebo	Generally manageable AEs; monitoring required
Sirolimus (Preventive)	mTOR (TSC)	Phase I trial	16 infants	Superior efficacy vs untreated historical cohort	Safe and tolerable in infants

Abbreviations: AAV, adeno‐associated virus; AEs, adverse events; ASO, antisense oligonucleotide; mTOR, mammalian target of rapamycin; RCT, randomized controlled trials; SCN1A, sodium channel, voltage‐gated, type I α subunit; SCN1B, sodium channel, voltage‐gated, type I β subunit; TSC, tuberous sclerosis complex.

## CONCLUSION AND PROSPECTS

Recent advances in pediatric epilepsy management have substantially transformed therapeutic paradigms; however, challenges remain. From a pharmacological perspective, third‐generation ASMs demonstrate improved efficacy and tolerability, but drug resistance represents a major obstacle. Surgical interventions provide promising options for drug‐resistant cases. Concerns regarding long‐term durability and accessibility disparities, especially in low‐resource settings, warrant further optimization. The ketogenic diet offers effective short‐term seizure control but poses metabolic risks with prolonged use, which require careful clinical judgment. Emerging disease‐modifying therapies hold transformative potential by targeting underlying etiologies. Clinical translation requires rigorous validation through well‐designed trials. Ultimately, the most appropriate and individualized treatment strategy should be determined via comprehensive consideration of etiology, epilepsy classification, and patient‐specific factors.

**FIGURE 1:**
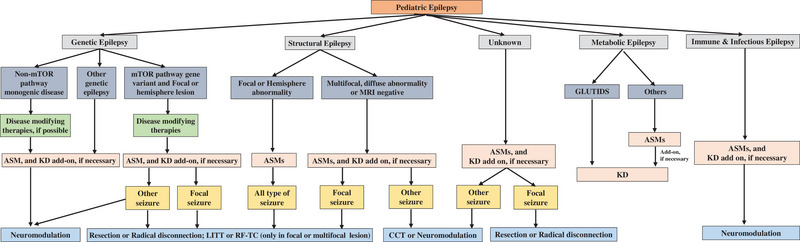
Treatment selection based on etiology and epilepsy classification. The therapeutic strategy prioritizes etiology‐specific interventions when available. The pathway begins with the identification of a treatable etiology (gray box). For patients without a targetable etiology or who are not candidates for curative/disease‐modifying therapy, symptomatic management with anti‐seizure medications and dietary therapy (if necessary) (pink box) is recommended. For patients with drug‐resistant epilepsy, surgical treatment (blue box), including resection surgery, disconnection surgery, ablation therapy, and neuromodulation, should be taken into consideration. ASMs, anti‐seizure medications; CCT, corpus callosotomy; GLUT1DS, glucose transporter type 1 deficiency syndrome; KD, ketogenic diet; LITT, laser interstitial thermal therapy; mTOR, mammalian target of rapamycin; RF‐TC, radiofrequency thermocoagulation.

Among the major treatment modalities discussed, no single approach is universally optimal for all patients with epilepsy; treatment selection should be individualized. Etiology‐targeted interventions directly address the underlying cause and should be prioritized in appropriately selected patients. Among individuals who are not candidates for etiological treatment, ASMs typically represent first‐line therapy. Ketogenic diet and neuromodulation are applicable to many patients with refractory epilepsy. These latter approaches are primarily symptomatic and reduce seizure frequency and/or severity without modifying disease mechanisms. Additional clinical factors must be integrated to formulate individualized treatment plans (Figure 1).

Future research should prioritize personalized medicine by integrating genetic, electrophysiological, and neuroimaging data to guide therapeutic selection. Standardized criteria for surgical candidacy and postoperative ASM management are essential to optimize neuropsychological recovery. Global collaboration will be critical to reduce inequities in access to care and establish large‐scale registries for rare epilepsy syndromes. By addressing translational gaps and promoting multidisciplinary innovation, sustained seizure control and improved quality of life for pediatric patients may be achieved.

## CONFLICT OF INTEREST

The authors declare no conflict of interest.
